# SHEEP AS AN EXPERIMENTAL MODEL FOR BIOMATERIAL IMPLANT EVALUATION

**DOI:** 10.1590/1413-785220162405161949

**Published:** 2016

**Authors:** SUELEN CRISTINA SARTORETTO, MARCELO JOSÉ UZEDA, FÚLVIO BORGES MIGUEL, JHONATHAN RAPHAELL NASCIMENTO, FABIO ASCOLI, MÔNICA DIUANA CALASANS-MAIA

**Affiliations:** 1. Universidade Federal Fluminense, Faculdade de Odontologia, Programa de Pós-Graduação em Odontologia, Niterói, RJ, Brazil.; 2. Universidade Federal do Recôncavo da Bahia, Centro de Ciências da Saúde, Santo Antônio de Jesus, BA, Brazil.; 3. Universidade Federal Fluminense, Faculdade de Veterinária, Niterói, RJ, Brazil.

**Keywords:** Biocompatible materials, Animal experimentation, Dental implants, Sheep, Orthopedic fixation devices.

## Abstract

**Objective:**

Based on a literature review and on our own experience, this study proposes sheep as an experimental model to evaluate the bioactive capacity of bone substitute biomaterials, dental implant systems and orthopedics devices. The literature review covered relevant databases available on the Internet from 1990 until to date, and was supplemented by our own experience.

**Methods:**

For its resemblance in size and weight to humans, sheep are quite suitable for use as an experimental model. However, information about their utility as an experimental model is limited. The different stages involving sheep experiments were discussed, including the care during breeding and maintenance of the animals obtaining specimens for laboratory processing, and highlighting the unnecessary euthanasia of animals at the end of study, in accordance to the guidelines of the 3Rs Program.

**Results:**

All experiments have been completed without any complications regarding the animals and allowed us to evaluate hypotheses and explain their mechanisms.

**Conclusion:**

The sheep is an excellent animal model for evaluation of biomaterial for bone regeneration and dental implant osseointegration. From an ethical point of view, one sheep allows for up to 12 implants per animal, permitting to keep them alive at the end of the experiments. Level of Evidence II, Retrospective Study.

## INTRODUCTION

The technical and scientific advances made in recent decades have enabled the development and improvement of new health technologies, mainly regenerative techniques and biomaterials. In this context, it is necessary to conduct experimental studies to evaluate the effectiveness of these techniques, as well as the cytotoxicity and biological behavior of these materials.

Experimental animal models have been widely used in biomedical research, because *in vitro* models are not able to fully mimic the human body's complexity.^1^ The animal models allow the development of basic knowledge that supports pre-clinical and clinical studies.^2^ Among the objectives for conducting research on animals are the collection of relevant information to understand the biology and wellbeing of humans and animals and the improvement and maintenance of health.^3^

With the current focus on animal preservation in experimental studies, research proposals should consider the replacement of animals with validated alternative methods, and the animals' wellbeing should be the primary consideration in the provision of care at all stages of the experiment^3^. Different animal models can be used for pre-clinical studies.^4^ ^-^ ^6^ However, the model must be carefully chosen to ensure the analogy to humans, to avoid excessive use of animals and to prevent the waste of time, effort and resources.^2^

Sheep are large animals suitable for medical research due to their availability and acceptance by the animal research society.^7^ However, the scientific literature using sheep as an animal model, particularly in dentistry and orthopedics, is limited regarding the handling and experimental surgical procedures.^8^ ^,^ ^9^ This article, grounded in our own experience and on the literature, reviews the advantages and disadvantages of using sheep for research as well as their breeding and maintenance, anesthetics, and pre-, trans- and postoperative procedures. The different stages involved in sheep experiments were discussed, highlighting the unnecessary euthanasia of animals at the end of study, in accordance with the guidelines of the 3Rs Program (Reduction, Refinement and Replacement).

## METHODS

The literature review was performed using the most important databases available on the Internet (PubMed, SciELO, LILACS, MEDLINE, and Cochrane databases) from 1990 to the present. The key words used were as follows: sheep, sheep animal model, experimental animal model, and implant system. A description of the experimental technique developed by our surgical research group from 46 experimental surgeries on 26 sheep in the last five years at the Fazenda-Escola da Faculdade de Veterinária, Universidade Federal Fluminense, Rio de Janeiro, RJ, Brazil, previously approved by the local Ethics Committee on Animal Use, was also presented.

### Review of the literature

The development of new biomaterials requires *in vitro* and *in vivo* experimental studies prior to pre-clinical and clinical use. *In vitro* analyses provide information about the cytocompatibility and toxicity of the biomaterial^10^ and about cell behavior when in contact with the biomaterial. This step avoids unnecessary animal use when the biomaterial tested is cytologically inappropriate.^10^ On the other hand, *in vivo* studies are able to evaluate the biocompatibility and the regenerative capacity of biomaterials, including the osseointegration of dental implants system and devices for fixation of bone fragments in the short and long term and in different types and qualities of bone tissue.^10^ ^,^ ^11^

The different animal models are divided into small animals (mice, rats and guinea pig), medium animals (rabbits, cats, mini-pigs and dogs) and large animals (horses, sheep and goats). When choosing the animal, researchers should take into account the following criteria: 1) ethical principles; 2) the ease and adaptability to experimental manipulation; 3) the cost and availability; 4) the possibility to study biological phenomena or animal behavior; 5) the investigation of spontaneous or induced tissue response; 6) the genetic uniformity among animals, where applicable; 7) the extrapolation of results for other animals or humans^12^; 8) the resistance to infections and disease; 9) the life span of animals in comparison with the duration of the study; and 10) the ability to develop experiments that mimic the clinical situation in humans.^11^

While bone healing of many animal species is recognized to be faster than in humans, sheep are considered to have a bone healing rate similar to humans^2^ and have been previously established as useful models for human bone turnover and remodeling activity.^12^ Based on this facts, sheep are suitable animals for use in experimental studies, primarily in studies that evaluate orthopedic and dental implant systems, and present advantages and disadvantages, compared to other experimental models. (Table 1)

**Table 1:** Advantages and disadvantages of using sheep as experimental models.

Advantages	Disadvantages
Acceptance by the animal research society^7^	Infrastructure of work and animal maintenance is more expensive^14^
Easy management^7^	Monitoring of vital functions by a veterinarian during all surgical procedures^14^
Body weight similar to humans^9^	Significant differences in the microstructure and density of bone tissue, depending on the site examined, compared to humans^11^
Composition, metabolism and bone remodeling similar to humans^9^ ^,^ ^13^	Limited availability of specific reagents to conduct immunohistochemical and *in situ* hybridization analysis^7^
Long bones with dimensions suitable for the deployment of implant systems and devices of bone fixation that are designed for humans^9^	
Excellent animal model to study osteoporosis due to long bones^13^	
Suitable to study the main physiological systems: cardiovascular, endocrine, respiratory, renal and reproductive systems^14^	
Enable evaluation of up to 12 implants (in the final size for marketing) per animal^15^	
Regeneration time similar to humans^2^ ^,^ ^9^ ^,^ ^11^	

### Breeding and maintenance of sheep for use as an experimental model

Surgical procedures in animals can only be developed after approval from the Ethics Committee on Animal Use at the institution where the experiments are performed. The animals selected for research should be properly developed, well nourished and healthy. They need to receive vaccines against infectious diseases according to the local needs of each region and country. The animals used in our group were vaccinated against rabies, clostridia, leptospirosis and infectious lymphadenitis, among others. 

In the breeding and maintenance of sheep, sufficient floor space is required for all sheep to be able to lie down at the same time in a normal resting posture, to adjust their posture, to move freely around and seek a comfortable location to rest and ruminate. Researchers must ensure that sheep have sufficient access to food (including salt and minerals) and water of adequate quality and quantity to fulfill their nutritional and physiological needs.^13^

When in confinement, ventilation systems ensuring adequate airflow to avoid excessive heat are necessary to minimize the risk of heat stress. Sheep should be provided with an appropriate period of rest without from artificial lighting (e.g., 6 hours), but they must not be kept in permanent darkness.^14^

Sheep must not be housed on solid concrete floors without providing adequate bedding. Various materials can be used as bedding for sheep, such as straw, wood shavings, paper products, peat and hemp.^13^

The animals used by our group were provided by the sheep sector at *Fazenda-Escola da Faculdade de Veterinária, Universidade Federal Fluminense*, Cachoeira de Macacu, Rio de Janeiro, RJ, Brazil. On this farm, the animals are maintained in semi-extensive system in paddocks containing native forage and Brachiaria forage (*Brachiaria humidicola* and *Brachiaria decumbens*). According to our protocol, these animals receive food composed of the above pastures in the preoperative period, and, postoperatively, in addition to food composed of pasture, they were also given sheep suitable nutritional supplements. Throughout the experimental period, mineral salt and water were provided *ad libitum*. Sheep reach sexual maturity between the seven and twelve months of life,^11^ and the growth of the distal femur and proximal tibia cease at 18-26 months of age. 

### Recommendations for achievement of surgical procedures for biomaterials and implant systems implantation

According to Biocompatibility Evaluation of Medical Devices International Standard Organization (ISO) 10993, up to 6 materials and/or implants can be installed on each sheep leg in the long bones (femur and tibia), amounting to twelve samples in a single animal. These cylindrical implants should not exceed 4mm diameter and 12mm in length and, in the case of screws-type implants, the dimensions must be 2 to 4.5 mm, respectively.^15^

### Pre-anesthetic Preparation and Anesthesia

All surgical procedures that can result in anxiety and/or pain to animals should be performed under general anesthesia. Ruminants, including sheep, can produce a higher volume of saliva when they are under anesthetic effect. This can be controlled by the administration of anticholinergic drugs, which avoid trans-surgical complications from airway obstruction. Another important feature to be observed during the anesthesia and trans-operative period of sheep is the possibility of regurgitation and aspiration of rumen contents, which may lead to suffocation or pneumonia. Using an endotracheal tube for pulmonary ventilation can help to avoid these incidents. If this is not feasible, we recommend keeping the head of the animal positioned so that the larynx is relatively high relative to esophagus.^16^

According to Gallates,^16^ before the anesthetic protocol, the animals need to be kept under food and water restriction. In studies performed in our group, the animals were deprived of solid and liquid food for 24 hours and 6 hours before anesthesia, respectively.^6^ ^,^ ^17^

**Figure 1:**
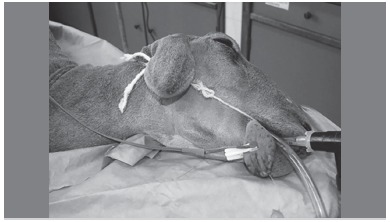
The sheep under general anesthesia and the endotracheal tube for pulmonary ventilation.

The anesthetic protocol used in our group to test biomaterials and implant systems consists of premedication with acepromazine (0.05 mg/kg, i.v., Acepran^(r)^, Vetnil, Louveira, SP, Brazil) and diazepam (0.2 mg/kg, i.v., Diazepam, Teuto, Anápolis, GO, Brazil) and morphine (0.4 mg/kg, i.m., Dimorf^(r)^, Cristália, Itapira, SP, Brazil) twenty minutes before the surgical procedures to reduce vagal tone. After observing the absence of pain reflexes, cephalic vein cannulation was performed and lactated Ringer's solution was administered (5 ml/kg/h, i.v., Baxter Hospitalar Ltda, São Paulo, SP, Brazil). Then, the animals receive intravenous anesthetic Propofol (4 mg/kg, i.v., Diprivan^(r)^, AstraZeneca, Cotia, SP, Brazil). The animals were intubated (orotracheal intubation) with a flexible tube and Cuff inflation, (Figure 1) and the general anesthetic was maintained by inhalation of 1% isoflurane (1% isoflurane^(r)^, Cristália, Itapira, SP, Brazil). After starting the anesthetic, lidocaine (4 mg/kg, Xylestesin^(r)^,Cristália, Itapira, SP, Brazil) and morphine (0.1 mg/kg, Dimorf^(r)^, Cristália, Itapira, SP, Brazil) are used for the epidural block. (Figure 1)

### Shaving and antisepsis

The sheep to be operated on was shaved before any antiseptic procedures. Antisepsis of the surgical area was achieved with degerming chlorhexidine and 2% alcoholic chlorhexidine. We suggest using a colorless solution to avoid animal distress by seeing a strange color on itself, such iodine solutions. Finally, properly sterilized surgical fields were placed for isolating and defining the surgical regions.

### Surgical procedures in tibia of sheep

**Figure 2:**
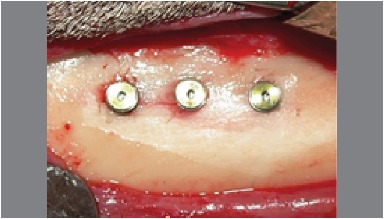
Dental implants in sheep tibia with a minimum of 2 cm distance separating each other.

An incision of approximately 5 cm in length along the long axis of the tibia was made with scalpel #3 and blade #15. When the bone tissue was exposed, the perforations were made with a minimum separation distance of 2 cm. The bone perforation was made with a 2 mm first drill (lance) to break the cortical bone tibia. After this step, a sequence of drills with increasing diameters was used according to the manufacturer's instructions of each implant system. To maintain the refrigeration of the surgical site and avoid local tissue necrosis by overheating, 1200 rpm were used under profuse irrigation with a 0.9% sodium chloride solution. (Figures 2 and ^3^) 

**Figure 3:**
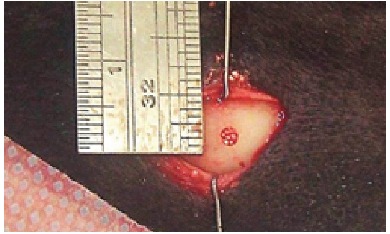
Biomaterial inside the sheep tibia after perforation.

After implant installation, the periosteum and soft tissue around the bone perforations were then placed back in position and attached to the subcutaneous tissue using an interrupted suture. The skin was closed using continuous #5.0 nylon sutures.

### Post-operative care

Postoperatively, the animals were kept in individual stall for 30 days and receive water *ad libitum* and chow and chopped forage twice a day. Each animal was confined in a 1.5 x 2.0 m stall lined with wood shavings for bedding. 

The wounds were kept uncovered and the operated tibia received no external immobilization. The post-operative pain and inflammation were controlled with tramadol (4 mg/kg, for oral Tramal^(r)^ Pfizer, São Paulo, SP, Brazil) and meloxicam (0.5/kg, for oral Meloxivet^(r)^ Duprat, Rio de Janeiro, RJ, Brazil) for 3 days. During the first post-operative week, antibiotic prophylaxis was administered using oxytetracycline (0.1 mg/kg/i.m., Terramicina^(r)^, Pfizer, São Paulo, SP, Brazil). Silver spray was topically administered daily to prevent local infection. 

### Surgical procedures for obtaining specimens and keeping the animal live 

**Figure 4:**
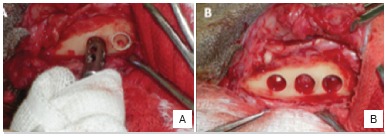
A) Bone blocks obtained by trephines under constant irrigation. B) Surgical cavities after collecting the samples.

After the experimental period, the animals were subjected to anesthetic procedures, as described before. All surgical areas were clipped, prepared and draped using a sterile technique. The skin incision was made to detach the periosteum and to expose the bone with the implant. The bone blocks carrying the implants were removed with trephines with a compatible diameter to that of the implant (keeping a 1 mm safety margin) under constant irrigation to prevent necrosis of the bone tissue. (Figure 4 A-B)

Since the animals were not euthanized at the end of experiment, they were taken to the stall for postoperative recovery. This step is similar to that performed after the first surgical procedure.

### Processing samples

**Figure 5:**
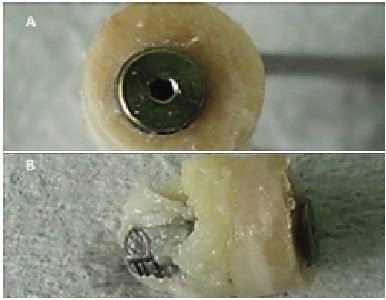
The obtained bone/implant sample.

The samples collected (Figure 5) were fixed in 4% formaldehyde buffer and embedded in resin (dental implants) or paraffin (biomaterials), and the slices were stained in toluidine blue or hematoxylin and eosin and examined with an optical microscope for histological and histomorphometric analysis or with a polarized light microscope.

## RESULTS

Until the present time, we have successfully carried out forty-six surgical procedures in three different studies. In order to conduct these studies, we followed the guidelines described in ISO Standard 10993-6,^15^ in which we evaluated the bioactivity and the bone repair capacity of bone substitute biomaterials and the interfaces between bone and different dental implants in different stages of osseointegration. We have experience using both genders of sheep. However, we have been careful to use the same gender of animals in each experiment in order to avoid biases. All animals were in good health during the healing period and did not show infection or any other post-operative wound-healing complications. All experiments have been completed without any significant untoward results for either the animals or the research staff and with positive results towards evaluating hypotheses and explaining mechanisms. (Table 2) 

**Table 2:** Table showing our experiments in which sheep as animal model were used. Note that no animal was euthanized at the end of experiments.

Study	Number of sheep	Gender	Implanted material	Size of defect/implant	Site	Experimental periods (days)	Postoperative conditions	Number of sheep euthanized
1	06	Female	hydroxyapatite and strontium-containing nanostructured hydroxyapatite	2.0mm diameter	Tibia	30	No complications	0
2	20	Female	Dental Implants	3.5mm x 7mm implants	Tibia	7. 14. 21. and 28	No complications	0
3	20	Female	Dental implants	2.9mm x 10mm implants	Tibia	7. 14. 21. and 28	No complications	0

## DISCUSSION

*In vivo* studies have greatly contributed to the understanding of the various physiological and pathological processes affecting humans.^18^ The careful choice of the experimental animal model depends mainly of the research objective, personal and institutional capacities, and the preference and experience of the researchers involved. In this context, the ethical and economic issues cannot be disregarded; instead, they should be well defined and standardized in order to reduce inter-study variations, allowing extrapolation based on reliable data and maximizing the validity of the obtained results.^16^

Useful data for clinical applications in humans should be based not only on good planning and design of the experimental study, but also on adequate knowledge of the animal used, taking into account the differences with humans.

Because there is no consensus on the ideal animal model and their anatomical, biochemical, physiological and biological characteristics similar to those found in humans, sheep have become a popular *in vivo* experimental animal model,^2^ mainly in orthopedic and dental research, due to the similarity of body weight, the presence of long bones and a bone regeneration time similar to humans'. These features allow researchers to conduct a proper evaluation of orthopedic and dental implants produced with dimensions for use in humans, allowing the extrapolation of results for use in humans with scientific credibility.^9^

The literature is limited regarding protocols for the surgical implantation of biomaterials and implants systems in sheep, as compared with small experimental models, for example, mice and rats.^4^ ^,^ ^19^ It is expected that the information contained in this article will assist in the achievement of pre-clinical studies through the extrapolation of data, which are possible due to the size of animal and size and long bones structure.^9^ ^,^ ^20^ ^-^ ^21^

## CONCLUSION

The sheep is an excellent animal model for the evaluation of biomaterials for bone regeneration and osseointegration of dental implant system in dentistry and orthopedics. Sheep are also compelling from an ethical point of view because according to the ISO, up to 12 implants can be tested per animal, and they can be kept alive at the end of the study, in accordance with the 3Rs principles.
